# miR-145 mediated the role of aspirin in resisting VSMCs proliferation and anti-inflammation through CD40

**DOI:** 10.1186/s12967-016-0961-2

**Published:** 2016-07-13

**Authors:** Xin Guo, Lijin Yu, Min Chen, Tian Wu, Xiangdong Peng, Ren Guo, Bikui Zhang

**Affiliations:** Department of Pharmacy, The Third Xiangya Hospital, Central South University, Changsha, 410013 Hunan China; Department of Pharmacy, The Second Xiangya Hospital, Central South University, Changsha, Hunan China

**Keywords:** Aspirin, miR-145, CD40, VSMCs, Proliferation

## Abstract

**Background:**

Aspirin (ASA) is the most widely used medicine to prevent cardiovascular diseases; however, the mechanisms by which ASA exerts its anti-proliferative effect remain not fully understood. This study was designed to investigate whether miR-145 is involved in the regulation of vascular smooth muscle cells’ (VSMCs) proliferation and to determine the anti-inflammatory effects of ASA via its regulation of CD40 to provide a new theoretical basis for the pharmacological effect of aspirin.

**Methods:**

The TNF-α induced proliferation model of VSMCs was divided into different groups with or without aspirin. Cell proliferation was detected by EdU; Real-time PCR was used to detect the mRNA expression of miR-145, CD40, and Calponin, a VSMCs differentiation marker gene. Western blot was used to detect the protein expression of CD40; ELISA was used to determine the concentrations of the inflammatory cytokine IL-6 in cell supernatants.

**Results:**

The proliferation of VSMCs was stimulated by TNF-α and accompanied by decreased levels of Calponin. TNF-α also decreased the levels of miR-145 and increased the levels of CD40 and IL-6. Pretreatment with 20 μg/mL of aspirin in VSMCs could partially block the above-mentioned effects induced by TNF-α. The protective effects of ASA in VSMCs were reversed by a pretreatment with a miR-145 inhibitor. We also found that the expression of miR-145 in peripheral blood mononuclear cells in ischemic stroke patients was significantly increased after a 10-day treatment with aspirin.

**Conclusion:**

miR-145 is involved in the anti-proliferation and anti-inflammation effects of aspirin on VSMCs by inhibiting the expression of CD40.

## Background

Atherosclerosis (AS) is the pathological basis of coronary heart disease and ischemic stroke (IS), which is characterized by abnormal proliferation of vascular smooth muscle cells (VSMCs) [1, 2]. The abnormal elevation of growth regulating factors, cell factors and vasoactive substances under pathological conditions can activate the VSMCs’ proliferation signal or inhibit their anti-proliferation signal, which in turn alter the gene expression profiles of VSMCs. In this case, the function and structure of VSMCs will be impaired and may cause them to migrate from the elastic plate into the endometrium and begin to proliferate. Excessive proliferation of VSMCs causes them to secrete a redundant extracellular matrix, which in turn causes the muscle tone in the cytoplasm of VSMCs to decrease and the number of organelles to increase, destroying the balance between proliferation and apoptosis.

miRNAs are short non-coding RNAs involved in the regulation of gene expression at the post-transcriptional level. miRNAs have been involved in the pathogenesis of a number of cardiovascular diseases. miR-145 is the most abundant miRNA in normal arteries and is mainly localized to VSMCs [3]. In dedifferentiated VSMCs, miR-145 is down-regulated and involved in the proliferation of VSMCs. In addition, miR-145 is a critical modulator of the VSMCs phenotype and proliferation [4, 5]. Overexpression of miR-143/145 promotes VSMCs differentiation and inhibits proliferation of VSMCs [6]. Boettger reported that the miR-143/145 gene cluster can regulate the contractile phenotype of VSMCs in miR-143/145-deficient mice [7]. miRNAs partially bind to complementary target sites in mRNA at the 3′UTR to regulate gene expression. Jakob and his colleagues have reported that miR-145 regulates VSMCs phenotype by targeting Krüppel-like factor 5 (KLF5) and helps control vascular neointimal lesion formation [3]. Other target genes, including Rho-associated, coiled-coil containing protein kinase 1 (ROCK1) and tripartite motif containing 2 (TRIM2), have also been identified as being controlled by miR-145 in cancer [8, 9].

CD40 belongs to the tumor necrosis factor receptor superfamily and is mainly expressed on the surface of B cells [10]. CD40 plays a critical role in T cell-dependent immune responses by interacting with the CD40 ligand (CD40L) [11]. The interaction between CD40 and CD40L can activate T and B lymphocytes and induce, for example, the expression of cytokines IL-1, IL-2, IL-6 and TNF-α [10]. In peripheral blood cells, CD40 and CD40L levels are significantly higher in patients with AS compared to normal controls [12]. CD40 and CD40L are also highly expressed in human atherosclerotic plaque, and the inhibition of CD40 signaling improves plaque stability [13]. These observations suggest that the expression of CD40 and CD40L may contribute to the formation of atherosclerosis.

Aspirin (ASA) is the most widely used medicine to prevent cardiovascular diseases and can inhibit CD40-CD40L ligation and reduce inflammation, alleviate atherosclerosis and stabilize plaque [14]. Plasma levels of sCD40L and TXA2 are elevated in atherosclerotic patients [15]. After administration of ASA to atherosclerotic patients, the release of sCD40L is significantly reduced via the suppression of the TXA2 generation [16]. As Riondino has reported, after being activated by some exogenous stimulus, sCD40L levels can be decreased by aspirin treatment both in vitro and in vivo [17]. As shown by previous reports, the interaction of CD40-CD40L induces VSMCs’ proliferation, whereas miR-145 and aspirin inhibits their proliferation [3, 18, 19]; however, whether the anti-proliferation effect of aspirin on VSMCs is mediated by a miR-145/CD40 dependent pathway remains unclear. In this study, we investigated the correlation between miR-145 and the anti-proliferative effect of aspirin to evaluate whether miR-145 is involved in the anti-proliferation and anti-inflammation effects of aspirin in VSMCs by inhibiting the expression of CD40.

## Methods

### Reagents

DMSO and TNF-α were purchased from Sigma (St. Louis, MO, USA). The miR-145 inhibitor and miR-145 inhibitor control were purchased from RiboBio Co., LTD (Guangzhou, China). Aspirin was obtained from the National Institutes for Food and Drug Control.

### Cell culture

Human aortic vascular smooth muscle cells were purchased from Jennio Biotech Co., Ltd (Guangzhou, China) and were cultured in Dulbecco’s modified eagle medium (DMEM) supplemented with 10 % fetal bovine serum (Hyclone, American), 100 U/ml streptomycin and 100 U/ml penicillin at 37 °C in a humidified atmosphere of 5 % CO_2_. VSMCs (3 × 10^5^) were transfected with a total of 200 nM of the miR-145 inhibitor or the miR-145 inhibitor control by using the Sofast transfection reagent (Xiamen, China) according to the manufacturer’s instruction. Total RNA and protein were extracted from VSMCs after transfection for 24 h, and RT-PCR was performed to measure Calponin or CD40 mRNA and miR-145 expression, while the expression of CD40 protein was evaluated by Western blot. The cell experimental groups were shown as follows: (1) DMSO: VSMCs were treated with a solvent as a blank control; (2) TNF-α: VSMCs were treated with TNF-α for 24 h; (3) +miR-145 inhibitor control: VSMCs were treated with TNF-α for 24 h after transfection with the miR-145 inhibitor control for 24 h; (4) +ASA: VSMCs were pretreated with aspirin for 1 h and then co-treated with TNF-α for 24 h; and (5) +miR-145 inhibitor +ASA: VSMCs were pretreated with aspirin for 1 h after transfection with the miR-145 inhibitor for 24 h. Then, VSMCs were treated with TNF-α for 24 h.

### Measurement of IL-6 levels

The concentrations of IL-6 in culture supernatants were measured by an ELISA Kit (Boster, China) according to the manufacturer’s instructions.

### Cell proliferation assay

Cell growth assays were performed with the EdU Kit (RiboBio, China) according to the manufacturer’s instructions. The proliferation of VSMCs showed red fluorescence under a microscope, and the ratio of EdU positive cell numbers was recorded to represent the VSMCs proliferation level.

### Real-time PCR

Total RNA was isolated with a Trizol reagent (TaKaRa, Japan) according to the manufacturer’s protocol. cDNA was prepared using the PrimeScript 1st Strand cDNA Synthesis Kit (TaKaRa, Japan). SYBR Green RT-PCR was performed using the following primers: Calponin: sense, 5′-AACCATACACAGGTGCAGTC-3′, antisense, 5′-GATGTTCCGCCCTTCTCTTAG-3′; CD40: sense, 5′- GCAGGCACAAACAAG ACTGA-3′, antisense, 5′-TCGTCGGGAAATTGATCTC-3′; GAPDH: sense, 5′-CTGCACCACCAACTGCTTAG-3′, antisense, 5′-AGGTCCACCACTGACACGT T-3′. For RT-PCR, amplification and detection were performed using the Real-time PCR Master Mix (ToYoBo, Japan) with the 7300 Real-Time PCR system (Applied Biosystems) according to the manufacturer’s protocol. The relative quantities were determined using the comparative CT method and were normalized to GAPDH for Calponin and CD40 mRNA and to U6 for miR-145 expression. All amplification reactions were performed in triplicate.

### Western blot analysis

The total protein concentration was determined using the BCA Protein Assay Kit (RiboBio, China). Proteins were denatured by boiling for 5 min, separated on SDS-PAGE gels, and transferred to a PVDF membrane. After blocking with 0.5 % milk for 1 h, the membrane was incubated overnight with mouse anti-CD40 (Santa Cruz, 1:1000), mouse anti-GAPDH (Biyuntian, 1:1000), followed by washing and incubating with goat anti-mouse (Biyuntian, 1:5000). Proteins were visualized by BeyoECL Plus Western blotting detection kit (Biyuntian, China), and pictures were captured using ChemiDoc XRS^+^ Image system (Bio-Rad, USA).

### Flow cytometry analysis

Cells were harvested, washed twice and resuspended with phosphate-buffered saline (PBS). A total of 100 μL VSMC suspension was added in each tube and stained with 5 μL of anti-CD40 antibody fluorescein isothiocyanate (FITC) for 30 min on ice in the dark. Then, cells were washed twice with PBS and were resuspended by 100 μL PBS. Flow cytometry analysis was performed within 2 h using BD FACSC antoII flow cytometer (BD Biosciences, USA) and data were analyzed using FlowJo software (Tree Star, USA).

### Subjects

Our study recruited 46 IS patients, all of which were enrolled at the Third Xiangya Hospital in Hunan, China from March 2015 to September 2015. All patients were Han Chinese, who lived in Changsha, China. IS was defined by focal neurological signs and was confirmed by brain CT and/or MRI using baseline conditions and brain CT using contrast medium after 48–72 h. The demographic characteristics of IS patients are shown in Table 1. The total RNA in the peripheral blood mononuclear cells (PBMCs) was extracted from all enrolled patients before and after the 10-day treatment with aspirin to determine the changes in miR-145 expression.

**Table 1:** General characteristics of the IS patients

Parameter	IS (n = 46)
Gender (male/female)	28/18
Age, year	56.23 ± 7.15
BMI, kg/m^2^	22.42 ± 2.81
SBP, mmHg	142 ± 15
DBP, mmHg	86 ± 8
Creatinine (Cr), μmol/L	85.56 ± 9.38
HDL-C, mmol/L	1.36 ± 0.32
LDL-C, mmol/L	2.53 ± 0.43
Triglyceride (TG), mmol/L	1.92 ± 0.49
Total cholesterol (TC), mmol/L	4.91 ± 0.82

This study was approved by the Ethics Committee of the Third Xiangya Hospital of Central South University (No. 2015-S099), and written informed consent was obtained from all subjects.

### Isolation of atherosclerotic plaque

The atherosclerotic plaques were obtained from AS patients using human carotid endarterectomy (CEA).

### Statistical analyses

Data are presented as the mean ± SD and were compared using a one-way ANOVA, followed by the Student–Newman–Keuls test. The difference of miR-145 expression before and after treatment with aspirin in IS patients was analyzed by using a paired t test. All analyses were conducted using the SPSS 13.0 software, and a value of P < 0.05 was considered to be statistically significant.

## Results

### miR-145 expression after transfection with miR-145 inhibitor in VSMCs

**Fig. 1:**
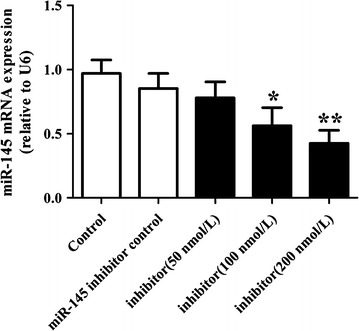
miR-145 expression after transfection with miR-145 inhibitor in VSMCs. The level of miR-145 expression in VSMCs was evaluated by Real-time PCR after transfection with different concentrations of the miR-145 inhibitor for 24 h. Values are presented as the mean ± SD; n = 3. Experiments were performed 3 times with similar results. *P < 0.05, **P < 0.01 vs control

The expression of miR-145 in VSMCs was determined by Real-time PCR after transfection with either miR-145 (50, 100, 200 nmol/L) or the miR-145 inhibitor control for 24 h. As shown in Fig. 1, the miR-145 inhibitor with 100 and 200 nmol/L concentrations inhibited the expression of miR-145. However, the inhibiting effect of the miR-145 inhibitor with the 200 nmol/L concentration was more significant. Then, VSMCs were transfected with miR-145 at a 200 nmol/L concentration in following part.

### miR-145 mediates the anti-proliferative effect of aspirin on VSMCs induced by TNF-α

**Fig. 2:**
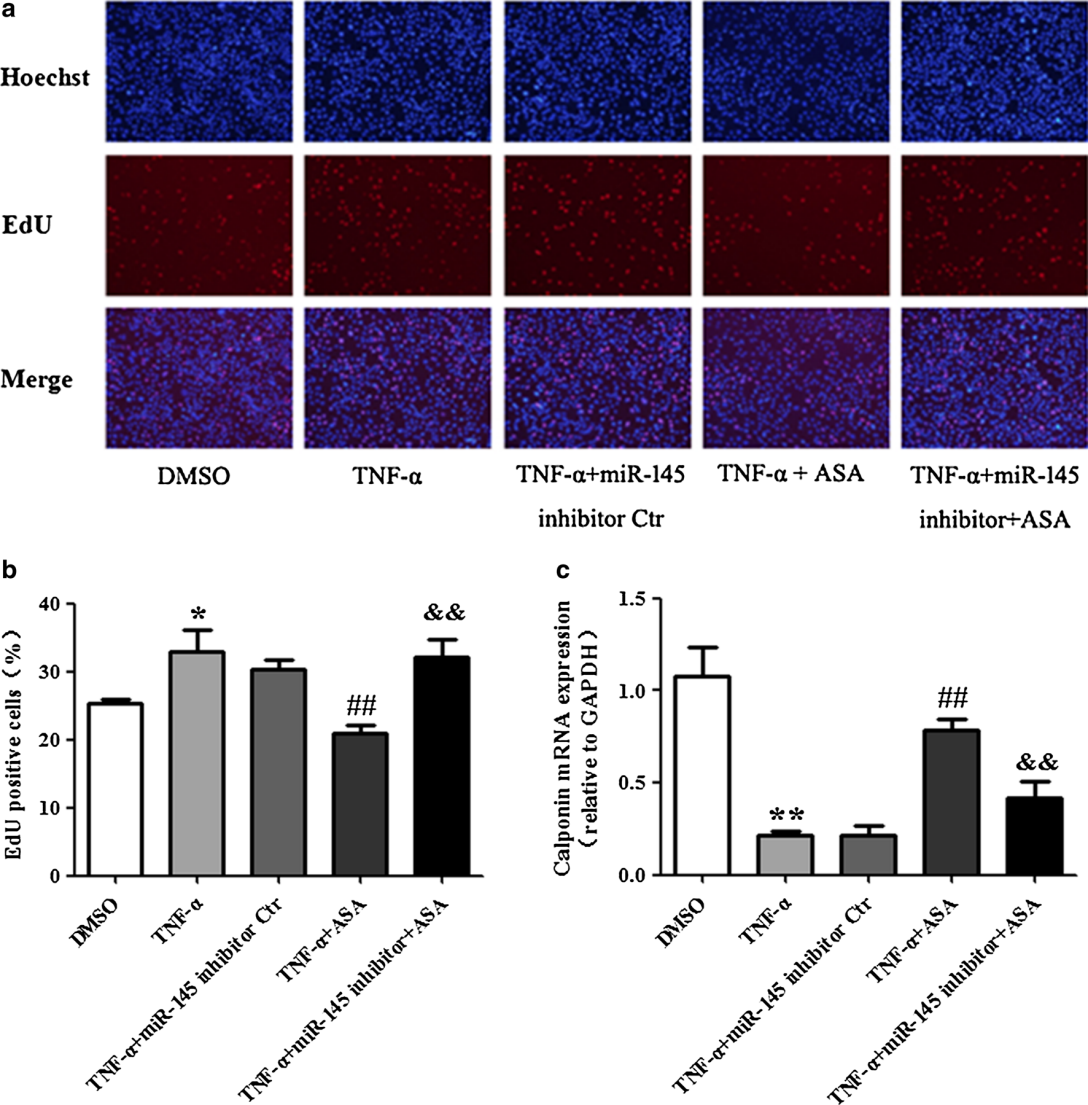
miR-145 mediates the anti-proliferative effect of aspirin in TNF-α treated VSMCs. VSMCs in different groups were treated as previously described. **a** The proliferation of VSMCs was determined by EdU; **b** statistical analyses of VSMCs proliferation; **c** the level of Calponin expression was determined by real-time PCR, and values are presented as the mean ± SD; n = 3. Experiments were performed 3 times with similar results. *P < 0.05, **P < 0.01 vs DMSO, ^##^P < 0.01 vs TNF-α, ^&&^P < 0.01 vs +ASA

**Fig. 3:**
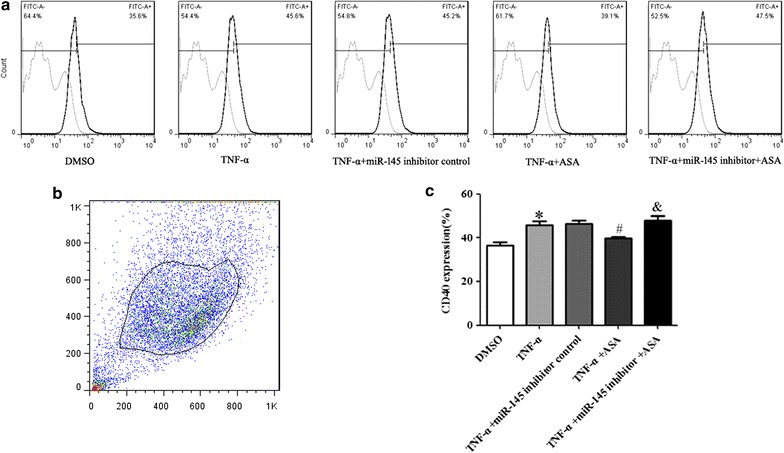
Evaluation of CD40 protein expression by flow cytometry inVSMCs. **a** CD40 expression on VSMCs with different treatment. **b** CD40 expression was determined by flow cytometry. **c** Statistical analyses of CD40 expression (%). Values are presented as mean ± SD; n = 3. Experiments were performed 3 times with similar results. *P < 0.05 vs DMSO, ^#^P < 0.05 vs TNF-α, ^&^P < 0.05 vs +ASA

Abnormal proliferation of VSMCs plays a role in pathological processes of vascular diseases, such as atherosclerosis, restenosis and hypertension [20–22]. In our research study, VSMCs proliferation and the anti-proliferative effects of aspirin in VSMCs were evaluated by EdU assays. As shown in Fig. 2a and b, VSMCs proliferation was induced in the TNF-α group. In the +ASA group, VSMCs proliferation induced by TNF-α was inhibited by aspirin. The miR-145 inhibitor counteracts the anti-proliferative effect of aspirin on TNF-α treated VSMCs. The mRNA expression of Calponin, a VSMC differentiation marker gene, was determined by real-time PCR. The Calponin mRNA level decreased when VSMCs were treated with TNF-α. Aspirin partly restored Calponin mRNA expression level. After transfection with the miR-145 inhibitor, the up-regulation of Calponin mRNA levels induced by aspirin was overturned (Fig. 2c). Similarly, the flow cytometry data of CD40 protein expression in VSMCs indicate that proliferating VSMCs cells induced by TNF-α were having higher expression of CD40 and upon treatment with ASA it reversed (Fig. 3a, b, c). These data suggest that the inhibition of miR-145 can reverse the effect of aspirin of up-regulating Calponin mRNA expression in TNF-α treated VSMCs and that miR-145 mediates the anti-proliferative effect of aspirin in TNF-α treated VSMCs.

### miR-145 mediates the effect of aspirin on CD40 expression induced by TNF-α

**Fig. 4:**
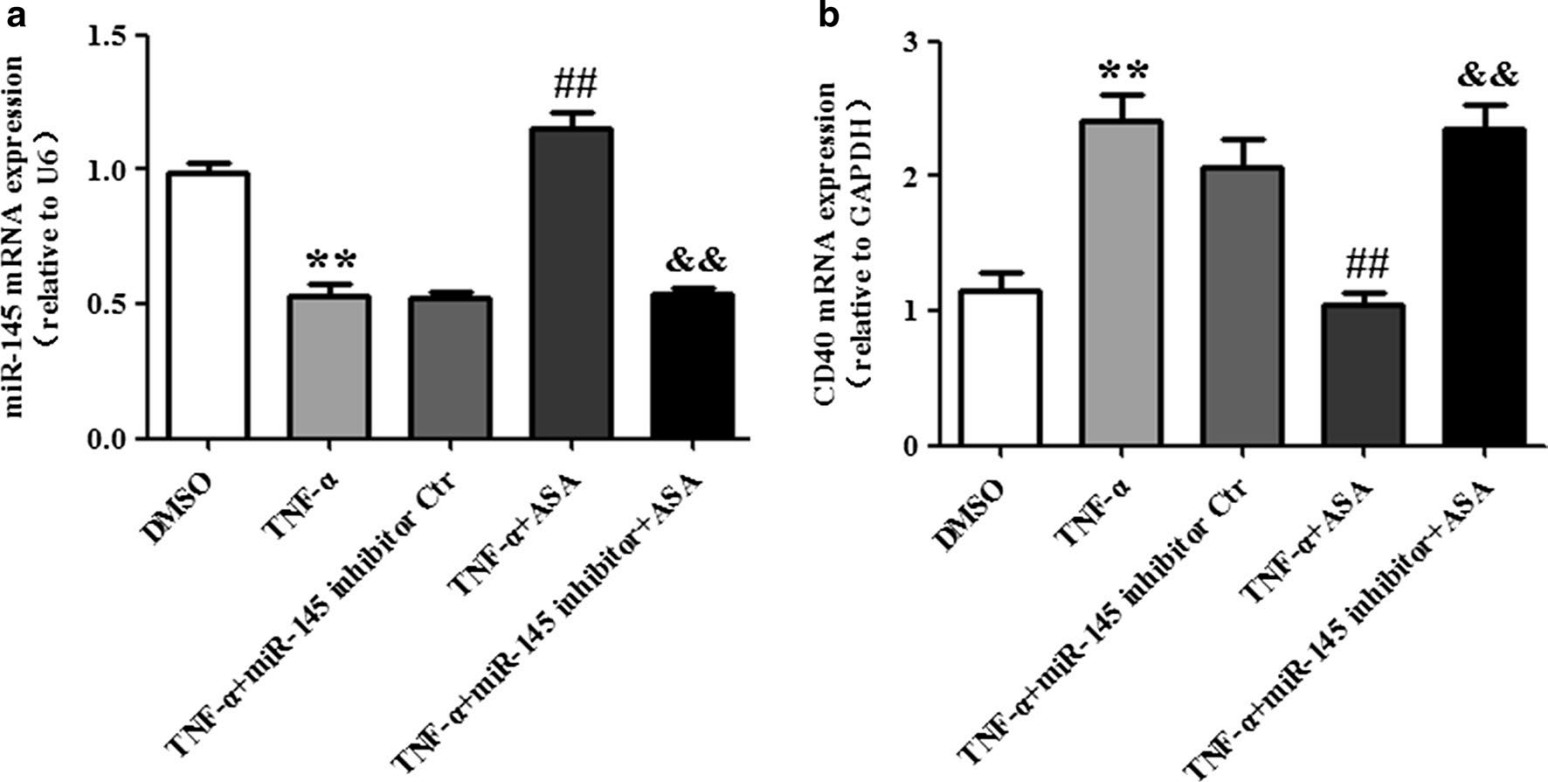
miR-145 mediates the effect of aspirin on CD40 expression induced by TNF-α. The levels of miR-145 expression (**a**) and CD40 mRNA expression (**b**) were determined by real-time PCR. Values are presented as the mean ± SD; n = 3. Experiments were performed 3 times with similar results. **P < 0.01 vs DMSO, ^##^P < 0.01 vs TNF-α, ^&&^P < 0.01 vs +ASA

**Fig. 5:**
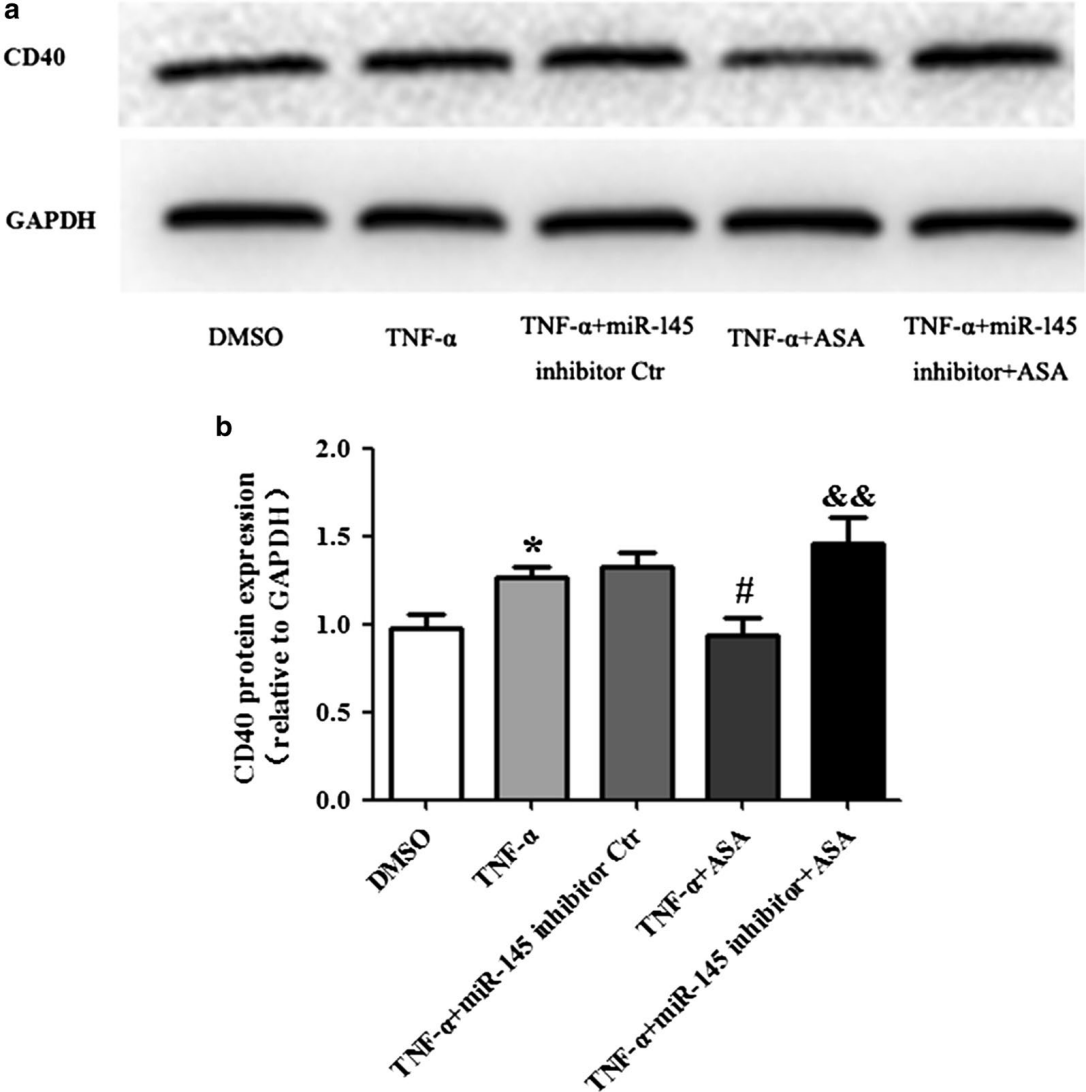
miR-145 mediates the effect of aspirin on CD40 protein expression induced by TNF-α. **a** The level of CD40 protein was determined by Western blot. **b** Statistical analyses of CD40 protein. Values are presented as the mean ± SD; n = 3. Experiments were performed 3 times with similar results. *P < 0.05 vs DMSO, ^#^P < 0.05 vs TNF-α, ^&&^P < 0.01 vs +ASA

**Fig. 6:**
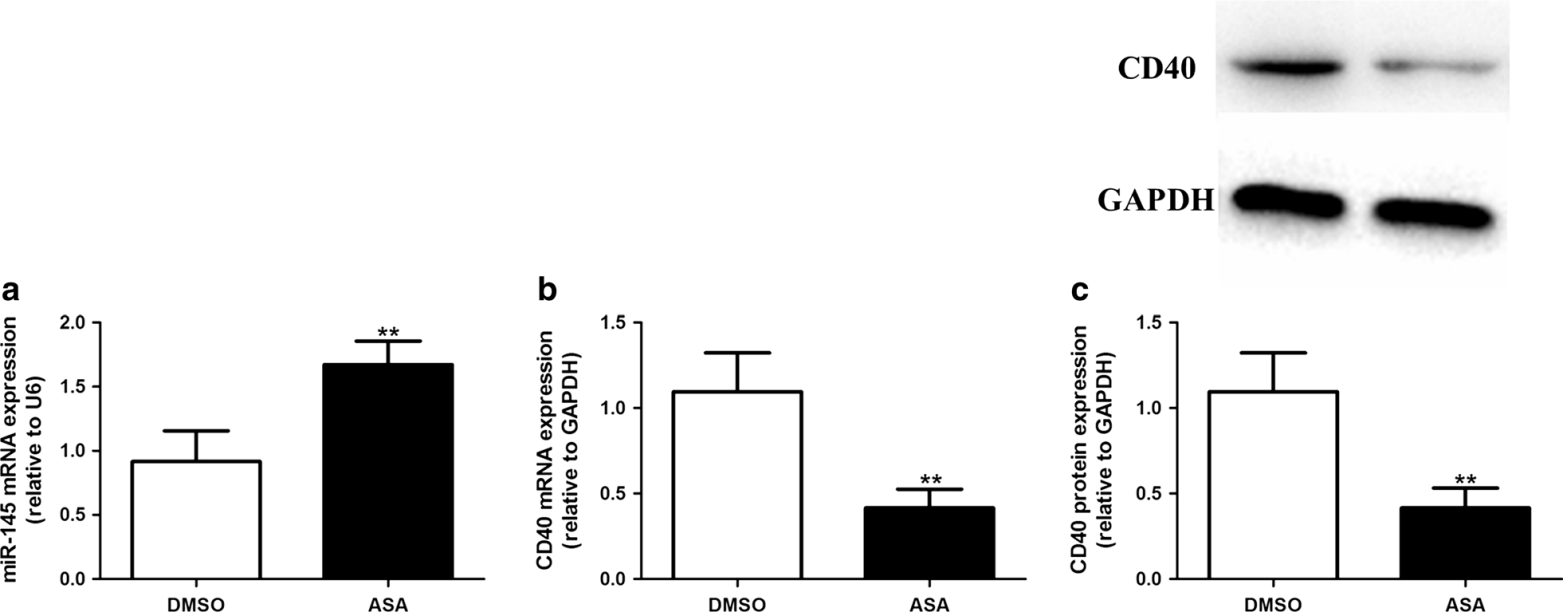
The effect of aspirin on miR-145 and CD40 expression in VSMCs. The levels of miR-145 expression (**a**) and CD40 mRNA expression (**b**) were determined by real-time PCR. **c** The level of CD40 protein was determined by Western blot. Values are presented as the mean ± SD; n = 3. **P < 0.01 vs DMSO

The levels of miR-145 and CD40 mRNA expression in VSMCs were determined by real-time PCR. Treatment of VSMCs with TNF-α significantly decreased the mRNA expression of miR-145 and increased the mRNA expression of CD40; whereas aspirin partially restored the mRNA level of miR-145 accompanied by a decrease in the mRNA level of CD40 compared to the TNF-α group. However, the effect of aspirin on miR-145 and CD40 mRNA expression was abolished in the +miR-145 inhibitor +ASA group compared to the +ASA group (Fig. 4a, b). When compared with the TNF-α group, the miR-145 inhibitor control did not show any significant effect on miR-145 and CD40 mRNA expression after TNF-α administration. The expression of CD40 protein was determined by Western blot. As shown in Fig. 5, CD40 protein expression is consistent with CD40 mRNA expression in every group. In addition, we explored the effect of aspirin alone in the absence of TNF-α on VSMCs and found that aspirin treatment significantly increased the miR-145 expression and decreased the CD40 expression both at the mRNA and protein level (Fig. 6a, b, c).

### miR-145 mediates the effect of aspirin on IL-6 induced by TNF-α

**Fig. 7:**
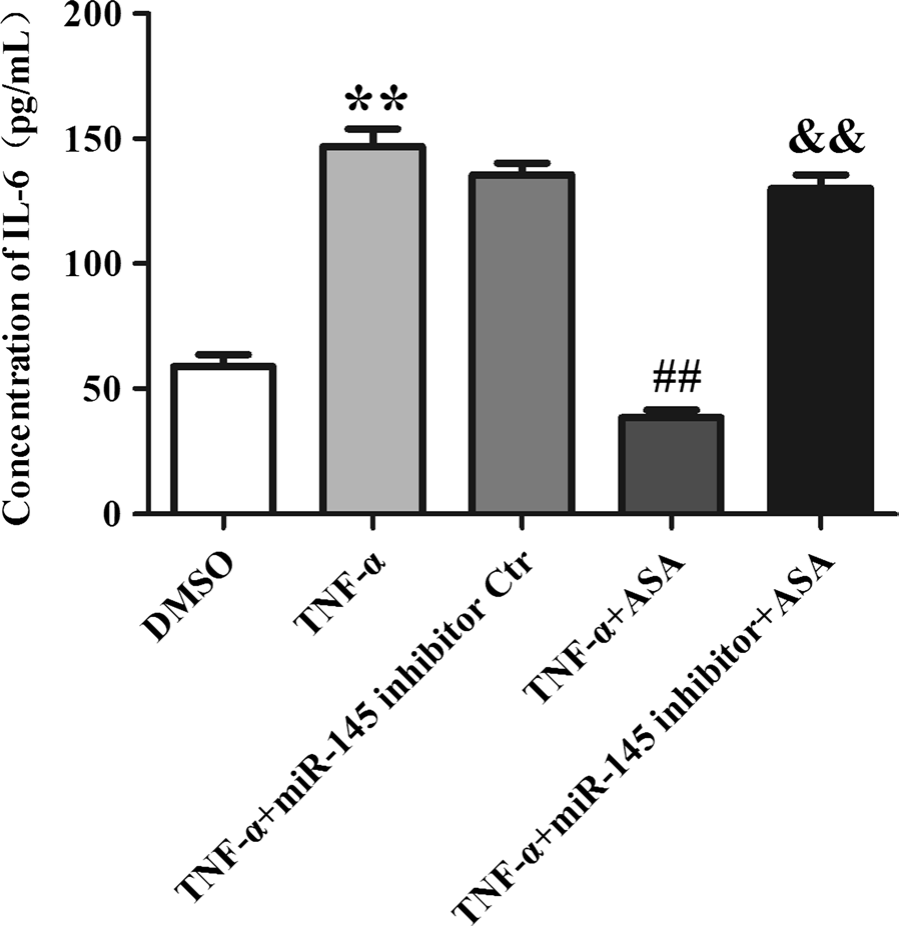
miR-145 mediates the effect of aspirin on IL-6 induced by TNF-α. Values are presented as the mean ± SD; n = 3. Experiments were performed 3 times with similar results. **P<0.01 vs DMSO, ^##^P < 0.01 vs TNF-α, ^&&^P < 0.01 vs +ASA

The pro-inflammatory cytokine IL-6 plays an important role in cell proliferation and is involved in the processes of many cardiovascular diseases. The concentrations of IL-6 in cultured cell supernatants were measured with an ELISA Kit. The IL-6 concentration was found to be higher in the TNF-α group than in the DMSO group. Treatment of VSMCs with aspirin partially decreased the IL-6 concentration induced by TNF-α. When compared with +ASA group, the effect of aspirin on IL-6 level was abolished by the miR-145 inhibitor in the +miR-145 inhibitor +ASA group (Fig. 7). Compared to TNF-α group, the miR-145 inhibitor control did not show any significant effect on IL-6 level induced by TNF-α.

### The influence of aspirin on the expression of miR-145 in IS patients

**Fig. 8:**
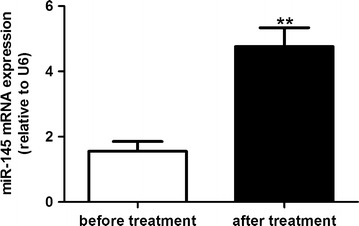
The influence of aspirin on the expression of miR-145 in IS patients. Values are presented as the mean ± SD; n = 46. **P < 0.01 vs before treatment

**Fig. 9:**
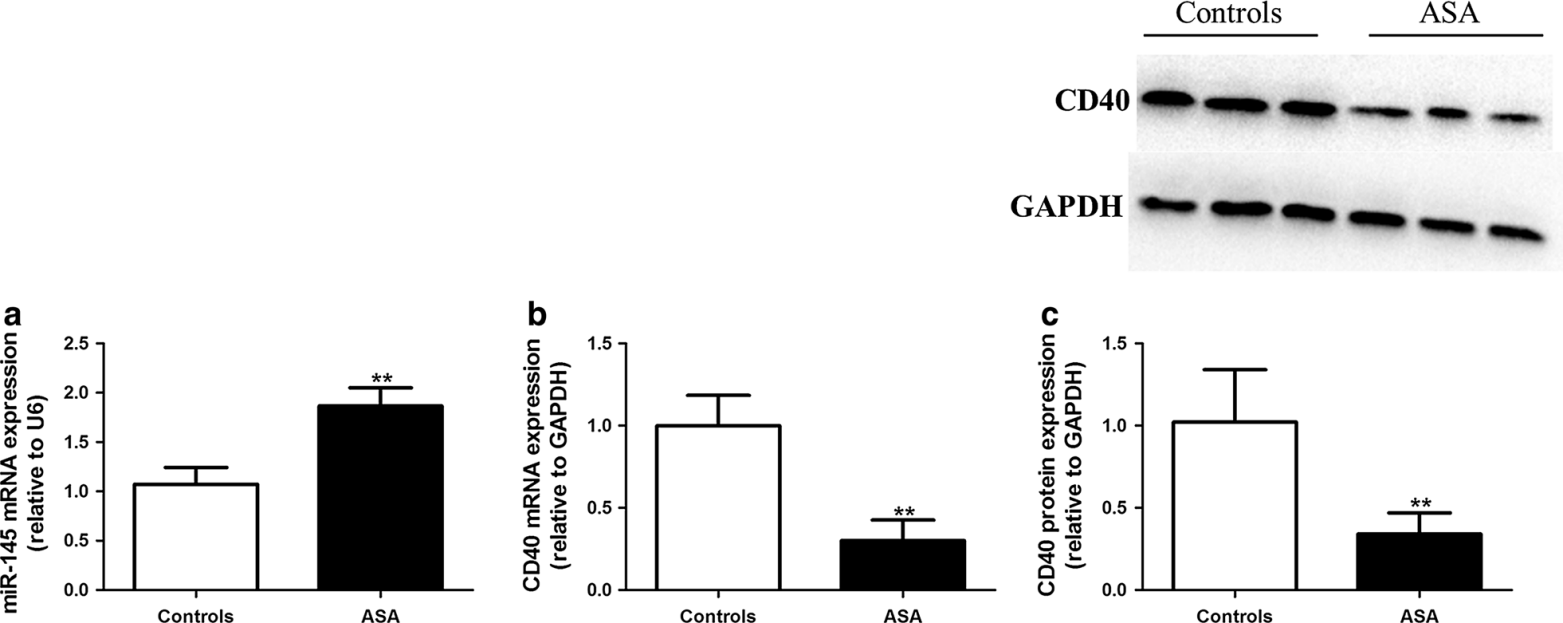
The difference of miR-145 and CD40 levels in AS patients with or without aspirin treatment. The levels of miR-145 expression (**a**) and CD40 mRNA expression (**b**) were determined by real-time PCR. **c** The level of CD40 protein was determined by Western blot. Values are presented as the mean ± SD; n = 21. **P < 0.01 vs controls

Aspirin is a commonly used drug for the treatment of IS. In this study, we used the stem loop primers of miR-145 synthesized by Guangzhou RiboBio Co. to amplify miR-145 in the PBMCs from IS patients. Our data revealed that the expressions of miR-145 were significantly elevated after the 10-day treatment of aspirin in IS patients, which suggested the increased expression of miR-145 in the IS patients may be partially induced by aspirin therapy (Fig. 8). Also, in this study, we collected several atherosclerotic plaque specimens from AS patients with or without aspirin treatment and found that the plaque from the patients treated with aspirin showed increased miR-145 levels and decreased CD40 levels (Fig. 9a, b, c).

## Discussion

VSMCs proliferation plays a critical role in the pathogenesis of various proliferative vascular diseases, such as atherosclerosis [23]. The inhibition of VSMC proliferation can be used as an approach to treating proliferative vascular diseases. TNF-α is produced in VSMCs and is involved in the production of cytokines, which can promote VSMCs proliferation and migration [24, 25]. Aspirin, a non-steroidal anti-inflammatory drug, has multiple pharmacological effects on inhibiting neointimal hyperplasia, thrombosis and proliferation. To confirm this role of aspirin in VSMCs, a group of cells was treated with aspirin for 1 h before being given TNF-α. We found that aspirin significantly inhibited the VSMC proliferation induced by TNF-α. We also found that when miR-145 expression was down-regulated by the miR-145 inhibitor, the inhibitory effect of aspirin on VSMCs proliferation was blocked. Calponin is a differentiation marker gene of VSMC. We used the mRNA expression of Calponin to evaluate the proliferation of VSMCs. The results of our study show that the regulatory effect of aspirin on Calponin mRNA expression needs miR-145 in accordance with the cell proliferation tested by the EdU assay. Above all, it is possible that the role of aspirin in suppressing cell proliferation is mediated by miR-145.

To further confirm the role of miR-145 in aspirin’s pharmacologically mediated effect, miR-145 expression was determined in VSMCs. The level of miR-145 expression decreased in the TNF-α group, and aspirin could up-regulate miR-145 expression, which was inhibited by TNF-α. Whereas the increase of miR-145 induced by aspirin disappeared upon intervention with the miR-145 inhibitor. This suggested that aspirin has an important role in regulating miR-145 expression. In agreement with the in vitro experiments, we also observed a definite association between aspirin and the expression of miR-145 in IS patients. Moreover, the atherosclerotic plaque from AS patients with aspirin treatment showed increased miR-145 and decreased CD40 levels compared to those from AS patients without aspirin treatment.

VSMCs can be induced to express CD40 by stimulation with TNF-α [26]. CD40L is involved in the process of inflammation, which mediates the progression of atherosclerotic disease. CD40-CD40L interaction also mediates plaque stability. In our study, CD40 mRNA expression was up-regulated by TNF-α and down-regulated by aspirin. Furthermore, the role of aspirin on CD40 mRNA expression was reversed with the miR-145 inhibitor. A similar trend was also observed in CD40 protein expression. Furthermore, there was a negative correlation between miR-145 and CD40 mRNA expression. These data showed that the effect of aspirin on CD40 expression induced by TNF-α was mediated by miR-145.

Inflammatory mediators, such as TNF-α and IL-6, can change VSMC survival and coronary plaque integrity [27]. The expression of IL-6 was elevated in serum and atherosclerotic plaques compared with that in the control [28, 29]. Previous researchers have demonstrated that IL-6 can induce vascular endothelial growth and is involved in regulating cell proliferation [30]. High levels of IL-6 have also been proved to be associated with cardiovascular diseases and the development of coronary disease in people without clinically established cardiac vascular diseases [31]. Emerging evidence has also identified a strong correlation between CD40-CD40L and IL-6 [32]. To determine the correlation between aspirin and IL-6 in VSMCs, we evaluated the levels of IL-6 in culture supernatants. As we have shown, aspirin significantly reduced the level of IL-6 induced by TNF-α, whereas the role of aspirin was mediated by miR-145.

## Conclusions

Our data indicate that aspirin significantly decreased the level of IL-6 in VSMC supernatants and suppressed VSMC proliferation and CD40 mRNA expression, as well as CD40 protein expression. When miR-145 expression was down-regulated by the miR-145 inhibitor, the effect of aspirin on VSMCs was reversed. This result suggested that miR-145 mediates the suppression of aspirin of CD40 induced by TNF-α. However, further research focusing on the role of miR-145 in inflammation or CVD/atherosclerosis model is still necessary to verify our findings. In conclusion, our study indicates that miR-145 is involved in the mechanism of aspirin’s inhibition of VSMC proliferation. These findings will help advance our understanding of the mechanism of aspirin’s inhibition of VSMC proliferation.
